# Assessing Diabetes-Relevant Data Provided by Undergraduate and Crowdsourced Web-Based Survey Participants for Honesty and Accuracy

**DOI:** 10.2196/diabetes.7473

**Published:** 2017-07-12

**Authors:** Mary Turner DePalma, Michael C Rizzotti, Matthew Branneman

**Affiliations:** 1 Ithaca College Department of Psychology Ithaca, NY United States

**Keywords:** crowdsourcing, diabetes mellitus, survey design, survey methodology, survey quality, mechanical turks, MTurk, data accuracy

## Abstract

**Background:**

To eliminate health disparities, research will depend on our ability to reach select groups of people (eg, samples of a particular racial or ethnic group with a particular disease); unfortunately, researchers often experience difficulty obtaining high-quality data from samples of sufficient size.

**Objective:**

Past studies utilizing MTurk applaud its diversity, so our initial objective was to capitalize on MTurk’s diversity to investigate psychosocial factors related to diabetes self-care.

**Methods:**

In Study 1, a “Health Survey” was posted on MTurk to examine diabetes-relevant psychosocial factors. The survey was restricted to individuals who were 18 years of age or older with diabetes. Detection of irregularities in the data, however, prompted an evaluation of the quality of MTurk health-relevant data. This ultimately led to Study 2, which utilized an alert statement to improve conscientious behavior, or the likelihood that participants would be thorough and diligent in their responses. Trap questions were also embedded to assess conscientious behavior.

**Results:**

In Study 1, of 4165 responses, 1246 were generated from 533 unique IP addresses completing the survey multiple times within close temporal proximity. Ultimately, only 252 responses were found to be acceptable. Further analyses indicated additional quality concerns with this subsample. In Study 2, as compared with the MTurk sample (N=316), the undergraduate sample (N=300) included more females, and fewer individuals who were married. The samples did not differ with respect to race. Although the presence of an alert resulted in fewer trap failures (mean=0.07) than when no alert was present (mean=0.11), this difference failed to reach significance: *F*_1,604_=2.5, *P*=.11, ƞ²=.004, power=.35. The modal trap failure response was zero, while the mean was 0.092 (SD=0.32). There were a total of 60 trap failures in a context where the potential could have exceeded 16,000.

**Conclusions:**

Published studies that utilize MTurk participants are rapidly appearing in the health domain. While MTurk may have the potential to be more diverse than an undergraduate sample, our efforts did not meet the criteria for what would constitute a diverse sample in and of itself. Because some researchers have experienced successful data collection on MTurk, while others report disastrous results, Kees et al recently identified that one essential area of research is of the types and magnitude of cheating behavior occurring on Web-based platforms. The present studies can contribute to this dialogue, and alternately provide evidence of disaster and success. Moving forward, it is recommended that researchers employ best practices in survey design and deliberately embed trap questions to assess participant behavior. We would strongly suggest that standards be in place for publishing the results of Web-based surveys—standards that protect against publication unless there are suitable quality assurance tests built into the survey design, distribution, and analysis.

## Introduction

### Study 1

#### Diabetes Self-Care

Diabetes is a complex, chronic illness in which a patient’s body has difficulty regulating the amount of glucose in the blood. This illness requires continuous self-care, which is critical to the prevention of acute and long-term complications. In 2013, the International Diabetes Federation estimated that 382 million people worldwide had diabetes, and that number is expected to increase to 592 million people by 2035 [1]. In the context of these projections, there is concern that the cost-heavy treatment of this disease may outstrip existing healthcare resources. Monies spent on the treatment of diabetes will then limit the funds available for the prevention of this disease, as well as the prevention of other chronic conditions.

As a result, research continues to investigate biological methods for treating diabetes. Because researchers estimate that 95% of care associated with the disease is personal behavioral self-care [2], research is also underway to examine the psychosocial markers of how well someone manages the disease. For example, DePalma et al found in a small, largely Non-Hispanic white sample, that greater perceptions of personal responsibility for disease onset were related to poorer diabetes self-care [3]. In a subsequent investigation of these variables in an American Indian and Alaska Native sample, DePalma et al failed to replicate this finding and instead found that diabetes self-efficacy was a strong predictor of more effective diabetes self-care [4]. Because of the possibility that racial, ethnic, or cultural differences played a role in these disparate findings, the researchers bore the responsibility of continued investigation on groups that are disproportionately affected by diabetes (eg, Asian Americans, African Americans, and Latinos). In order to eliminate health disparities, research will depend on our ability to obtain such select groups of people (ie, samples of a particular racial or ethnic group with a particular disease); unfortunately, researchers often experience difficulty recruiting samples of sufficient size [5].

#### The Need for Sample Diversification

There are obvious and practical reasons why the bulk of research is on undergraduates, but there has been a strident call to work toward sample diversification, particularly in health research. This concern is not new; the limitations of using undergraduate samples for conducting research have been discussed for decades. Given that this is particularly true in the social and behavioral sciences, Arnett evaluated the diversity of psychological research by analyzing 4037 studies from six different American Psychological Association journals published over 20 years [6]. Analyses showed that in 2007 alone, 67% of American studies published in the *Journal of Personality and Social Psychology* used undergraduate psychology participants. In countries other than the United States, undergraduates were used in 80% of studies [6]. Henrich et al estimated that when participants are selected for research, an American undergraduate is 4000 times more likely to be selected than is a non–Western individual [7]. Arnett argued that “the rich get researched” [6].

Of course, researchers should be cautious when extending results from undergraduate participants to diverse adult populations. Why would observations of samples of 18-22 year old undergraduates who are primarily white and increasingly female [8] be expected to generalize to phenomenon describing diverse health, business, and social behaviors? Using a series of large-scale meta-analyses, Peterson showed that, when compared with responses from non-student samples, undergraduate psychological and behavioral responses were more homogenous and the associated effect sizes often differed in magnitude and direction [9]. This could be especially problematic when investigating disparities that exist in a behavioral health context. Notably, Peterson and Merunka observed “...even if theory testing is the study purpose, few researchers using convenience samples of college students appear to recognize that their investigation possesses the characteristics of a limited laboratory test that cannot generalize to other samples” [10].

In addition to concerns about response homogeneity, some researchers have also questioned the quality of undergraduate data. Chen utilized data from the National Survey of Student Engagement (NSSE) involving undergraduates from 587 US colleges and universities [11]. About 11% of first year and 7% of fourth year undergraduates failed to answer 30% or more of the 85 Web-based survey questions. Students who responded to the Web-based version provided more responses of lower quality than did those responding in a paper-and-pencil format. Chen expressed concern that participants may not properly understand survey questions or that their responses may be careless, negatively affecting the quality of the resultant data.

#### Web-Based Samples Can Be More Diverse

Although the extensive reliance on undergraduate samples remains in practice, an increasing amount of survey research is now being conducted on the Internet, which allows researchers to quickly and easily collect data from local and global participants [12]. While there are certainly students on these survey platforms, a researcher is no longer restricted to samples of undergraduate psychology students.

#### Amazon Mechanical Turk (MTurk)

MTurk, a Web-based crowdsourcing platform for conducting survey research, is touted as providing an economical, diverse sample [13]. Based on their research, Crump et al dramatically conclude that Amazon MTurk (AMT) “...is a revolutionary tool for conducting experiments. It offers the ability to run experiments with large numbers of subjects in a matter of hours. This has the potential to transform behavioral research. Additionally, AMT provides an opportunity to reach a more representative population that varies widely in age, education, and ethnicity and geographic location” [14]. Amazon Mechanical Turk seemed to have the potential to fulfill our need to obtain a specific sample efficiently and inexpensively.

On MTurk, “Requesters” post Web-based Human Intelligence Tasks (HITs) to be completed by “Workers” who are paid to complete the HIT. There are typically more than 100,000 HITs that are readily available for MTurk Workers [13]. For example, HITs might include completing basic surveys or performing accounting tasks. An MTurk Worker then earns a HIT quality “approval rating” based on the number of HITs accepted by the Worker compared to the number of times Requestors reject the completed work for being of low quality. Accepted work then receives compensation ranging between US $.01 and several dollars per HIT. In essence, a survey researcher could conceivably collect 1000 responses from a 10 min survey in less than one week for US $100 [15]. It is easy to see how this rapid and inexpensive mode of data collection could be attractive.

The primary draw for our research team was the purported diversity of MTurk participant pools. Kraut et al contend that internet-based surveys “...can provide a large, diverse sample at low cost” [16]. Mason and Suri report that MTurk Workers “...tend to be from a very diverse background, spanning a wide range of age, ethnicity, socioeconomic status, language, and country of origin” [17]. MTurk includes more than 500,000 Workers from 190 countries [13], including the United States (47%) and India (34%) [18]. Of the US MTurk Workers, there are currently more women (64.85%) than men (35.15%), and many have a higher educational level than the general US population [18]. Although Berinsky et al report that MTurk samples are largely white in terms of racial composition, these samples are considered comparable to adult participants found in other convenience samples [19].

In addition, college samples are not an efficient option for conducting some types of health research because these groups tend to be too young to produce significant sample sizes of people with diabetes and other chronic illnesses. Thus, MTurk is likely to be superior to a college population whenever the researcher is examining health issues that are not widely present in undergraduate samples. Finally, although it is important to note that MTurk samples are expected to be more diverse because they would include anyone over the age of 18, be larger than one cohort of students, and extend beyond a single college campus, it is also important to emphasize that this would not necessarily result in more socioeconomically diverse samples. Individuals who do not have access to computers and the Internet will not be represented in these samples.

#### Web-Based Sample Quality

There is no conclusive answer regarding the quality of MTurk responses as the available data offer a conflicting report. Some evidence suggests that Web-based samples are of worse quality than undergraduate samples. Rouse reported that MTurk responses to a personality measure were less reliable than responses reported for an adult community sample [20]. MTurk Workers also tend to score slightly higher on social desirability [21]. The desire to please researchers may be detrimental because the Worker may provide the answer they believe the researcher wants or look to outside sources for more information to “correctly” answer questions [22,23]. Kees et al contend, however, that lower quality MTurk data is largely the result of using MTurk Workers who have non-US IP addresses [24].

In addition, some people have expressed concern that only a limited number of participants are accounting for a significant proportion of the data produced by MTurk Workers [25]. For example, Kumar suggests creating a reusable list of Workers who routinely provide high quality data [26]. Although this may be an excellent strategy for individual HITs that are using Workers for “work,” this would defeat any use of these pools for research conducted for the purpose of collecting generalizable data.

However, other research evidence suggests that Web-based samples produce data comparable to, or substantially better than, those obtained using traditional samples. However, there is the added benefit of potentially being more diverse [27]. For example, Paolacci et al compared the data collected on MTurk Workers to a traditional subject pool from a Midwestern US university [18]. MTurk Workers were not more likely to cheat than undergraduate participants, nor was there evidence suggesting that Web-based methods produced poorer quality data. The authors concluded that data collected through MTurk Workers can be comparable to data collected from more traditional means. Mullinix and colleagues compare population-based data and MTurk data across 20 studies and conclude that there is considerable similarity in treatment effects, supporting the potential utility of MTurk samples [28]. Clifford and Jerit provided even more striking data that showed that student samples self-reported cheating at rates between 24-41%, while comparable MTurk self-reports hovered between 4-7% [29]. The authors acknowledge, however, that it could be that MTurk respondents were less likely to report cheating behavior because of the impact such an admission might have on their approval rating or pay. However, in a direct comparison of MTurk and undergraduate samples, Hauser and Schwarz report that, across three studies, MTurk respondents were significantly more attentive to specific instructions contained in manipulation checks than were respondents from undergraduate subject pools [30].

#### Purpose

Based on previous research, we hypothesized that a judgment of responsibility for the onset of diabetes would be related to disease self-care. MTurk’s diversity attributes would offer a promising tool to examine this hypothesis in a large and diverse sample.

However, preliminary analysis of the data revealed significant inconsistencies. These irregularities spurred the investigation of the quality of our initial data for health-relevant material, and prompted a second direct test of the quality of MTurk data. 

### Study 2

#### Survey Design Practices

Of course, researchers bear the responsibility of being continually vigilant and cognizant of the quality concerns for all self-report data, independent of whether the participant is physically present or on the Internet. Even if care has been exercised in the recruitment of respondents, participants can occasionally subvert the onboarding process and contribute responses that would be unhelpful at best and misleading at worst. Maniaci and Rogge present evidence that poor quality data may reduce power and effect sizes and obscure findings that are visible in the responses of attentive respondents [31]. Thus, researchers could incorporate survey design practices to attempt to increase quality. Researchers can choose from attention checks or reminders, alerts, or actual trap questions; however, each of these methods has strengths and weaknesses.

#### Attention Checks

Goodman et al suggest the use of methods to gauge participant attention [32]. Attention filters are “trick” questions that require a respondent to answer in a particular way in order for the survey to continue; that is, the survey process does not continue until the “correct” box is checked. However, Paolacci and Chandler contend that having these types of attention checks are no more beneficial to having higher quality data than just working exclusively with Workers who have high approval ratings [22].

#### Alerts

Some research suggests that the use of a warning message or an alert will produce higher quality data. Clifford and Jerit found that the presence of an item asking respondents to be attentive and honest produced more reliable responses [33]. While some researchers have expressed concern that these types of affirmations may be interpreted negatively in light of a reference to participant honesty, Clifford and Jerit report that respondents were not visibly upset by their survey manipulation that specifically asked participants not to use outside sources to find a correct answer [29]. These “honesty affirmation” items may prod people to be more conscientious, but these items will not provide a way to evaluate whether participants were actually conscientious in their responses.

#### Trap Questions

Trap questions can be included within surveys to identify respondents who are not reading carefully (or at all) or who are using automated response methods. Examples of trap questions include simple requests to choose a specific answer from a subsequent response list. Or, the response of a participant who answered “yes” to being a biological male could be compared to his response on the question: “have you ever been pregnant?” Downs at al suggest that researchers should deliberately embed trick questions to measure whether participants answer conscientiously [34]. These “catch trials” would help researchers determine which subjects were not paying close attention.

#### Worker Qualifications

When creating a HIT, it is possible to manage the level of qualifications a Worker needs in order to be able to participate. For example, one could increase the approval rating to 95% and increase the required number of previous HITs that have been completed successfully. Another strategy is to restrict the survey to MTurk Masters, who are “...an elite group of Workers, who have demonstrated superior performance while completing thousands of HITs for a variety of Requesters across the Mechanical Turk Marketplace” [35].

#### Purpose

The potential for the conduct of research through the Internet is staggering. In fact, a 2011 article published in *Science* presented the MTurk platform as likely to become a “mainstream” form of data collection [36]. Published studies are now appearing that use MTurk participants; however, few provide information on the quality of the resultant data. Although there appears to be significant potential for MTurk to be a “revolutionary tool” that could assist in reaching more diverse samples, there remains significant concern over the quality of the resultant data as well as the degree to which these samples are truly diverse..

Therefore, the present study utilized both an undergraduate and an MTurk sample and hypothesized that the conscientiousness of the participants’ responses could be evaluated using trap questions as well as the time of survey completion. In addition, we randomly assigned half of the participants to receive an alert statement. We hypothesized that an alert statement would positively influence response quality. This study was designed to employ stronger restrictions and directly test whether MTurk can be a reliable data collection method for health-related information gathered from a diverse sample.

## Methods

### Study 1

#### Materials and Procedure

Subsequent to Institutional Research Board approval, a 25-min “Health Survey” was posted on MTurk. To investigate the psychosocial determinants of diabetes care in the United States, only US Workers who had a HIT approval rating of greater than 90% could “accept” the HIT, which allowed them to access the survey.

#### Qualification Questions

A preliminary qualification question required Workers to disclose whether or not they had any of the following diseases: diabetes, heart disease, asthma, osteoporosis, or none of the above. If diabetes was not selected, the participants were directed out of the survey and thanked for their interest. Workers who did select diabetes were prompted with a secondary age qualification question. Only Workers who specified that they were at least 18 years of age were allowed to continue to an informed consent page, where they again confirmed that they were 18 years of age or older, with diabetes.

#### Survey Questions

If the participants successfully met the relevant criteria, they completed a 40-question survey that included multiple choice, fill-in-the-blank, and Likert-type scale items. Workers first answered basic demographic questions (eg, age, sex, and race). Additional scales were included to measure psychosocial aspects of diabetes self-care. Participants were also prompted about their own disease status: “With which type of diabetes have you been diagnosed?” Answers included “type 1,” “type 2,” “I don’t know,” or “I don’t have diabetes.” At the conclusion of the survey, participants who entered a valid MTurk ID received a code to obtain their compensation of US $0.25. A debriefing statement was subsequently provided.

### Study 2

#### Participants and Procedure

A 10-min survey on “Health Issues and Health Organizations” was created using Qualtrics and, following Institutional Research Board approval, released on MTurk and the SONA systems platform (a local Web-based survey management system) for approximately 15 weeks. Restrictions were imposed such that only one response could be entered from a particular IP address. After providing informed consent, participants were directed to the survey, which ultimately concluded with a CAPTCHA and a debriefing statement.

##### Sample 1: MTurk

The survey was posted as a HIT available only to US MTurk Masters with a 95% approval rating over 1000 HITs. At the conclusion of the survey, participants who entered their MTurk IDs received a compensation of US $0.75.

On MTurk, 377 participants accessed the survey, but only 83.8% (316/377) of the participants finished the survey (138 were male, 176 were female, and 2 did not provide a response to this question). Demographic information for these samples can be found in Table 2. The individuals ranged in age from 20-69 years (mean=37.67, SD=11.83).

##### Sample 2: Undergraduate Sample

Undergraduates were recruited from introductory psychology classes to complete a Web-based SONA survey for which they received extra credit in a course. These undergraduates were recruited from non-majors courses and participants spanned the academic range from first-year students to seniors. Although 330 undergraduate participants accessed the survey, only 90.9% (300/330) finished the survey. Participants ranged in age from 17-62 (mean=19.37, SD=2.97; see Table 2).

#### Materials

##### Primary Measures

In a survey that was pre-tested to take less than 10 min, participants responded to basic demographic items (eg, age, sex, and race), an assessment of personal and family health history regarding different diseases (eg, diabetes and lung cancer), as well as their personal diabetes information (eg, type of diabetes, treatment, and medication).

##### Alerts

Approximately half of the participants were randomly assigned to an alert condition that examined whether an emphasis on conscientiousness would positively influence the quality of the participants’ survey responses. The alert was preceded by the word “IMPORTANT” in large bold red font, followed by the message “The following is a health survey that relies on your conscientiousness. We ask that you be attentive because your input will strengthen our understanding of an important area of research for the health community. Please also note that not being truthful breaches research (MTurk) guidelines. Thank you. We greatly appreciate your participation.”

##### Trap Questions

The Web-based survey included a total of 26 potential trap questions to measure participant conscientiousness. Participants were first asked to record the current date. They were also asked about family disease history. In a list of diseases, nine trap failures were embedded to check if responders claimed to have been diagnosed with, or had a family member diagnosed with, a fictitious disorder (eg, hyperemblyopia). There were 13 linked trap questions, which are those that are mutually exclusive. For example, if a participant identified his biological sex as “male” and responded “yes” to having been pregnant, trap failure would be noted. Finally, a trap failure would be recorded if participants noted at the beginning of the survey that they had diabetes and then later indicated that they did not, or indicated that they had both type 1 and type 2 diabetes.

##### Secondary Measures

As part of our cover story, we presented exploratory questions regarding health-related organizational “footprints.” These questions measured the participants’ knowledge and the perceived visibility of different health organizations. In addition, behavioral and lifestyle risk factors have been shown to be related to the onset of diabetes and heart disease (eg, sedentary living and poor dietary choices). Several studies have shown robust effects such that the perception that one could control disease onset will result in higher levels of perceived responsibility for disease onset, as well as higher ratings of blame [37]. To examine the ability to replicate these findings in the present sample, participants were also randomly assigned to evaluate a vignette that presented individuals with a disease (diabetes or heart disease) reportedly caused by either genetics or lifestyle choices. Using a Likert-type scale ranging from 1 (strongly disagree) to 5 (strongly agree), participants rated their emotional reaction toward the individual (ie, perceived responsibility, anger, and blame).

## Results

### Study 1

#### Participant Information

Using the MTurk platform, 4165 responses to the Health Survey were recorded over 6 months. However, initial data analyses revealed that an unusual number of data points had been entered in close temporal proximity from the same IP address. Further examination of this finding prompted a complete shift in the data analysis plan. The sample was abandoned for the original purposes, and we began a new focus on investigating the quality of the resultant data.

Of the original 4165 survey responses, 2667 responses (64.03%) came from individuals who made one attempt to take the survey, but did not meet the qualifications. Two hundred and fifty two individuals (6.05%) met the criteria for inclusion in the study. However, 1246 data points (29.92%) came from duplicate IP addresses. These 1246 data points had been entered by individuals coming from 533 distinct IP addresses (see Table 1). This subgroup of participants attempted to take the survey from two to six times (mean=2.34 attempts).

**Table 1:** Number of attempts per distinct IP address.

Number of attempts per IP address	n	Total number of responses
2	400	800
3	101	303
4	19	76
5	11	55
6	2	12
Total	533	1246

Of the 533 participants making repeated attempts, 48.0% (256/533) made multiple attempts reporting diseases other than diabetes. The remaining 277 participants (52.0%, 277/533) reported having diabetes in at least one of their attempts. Most of these participants (n=210) began taking the survey reporting diseases other than diabetes or no disease at all; they were subsequently excluded from the study. However, these participants returned moments later, after multiple attempts, to ultimately indicate that they had diabetes. Of these 210 participants, 185 participants “developed” diabetes within 60 s. A much smaller group (n=13) began the survey by indicating they already had diabetes, but later reported on a subsequent attempt that they did not have diabetes. However, it took much longer for these participants to be “cured” of their diabetes - approximately 3.57 h.

Had we not examined the data for the duplicate IP addresses, we would have simply restricted our sample to those respondents who indicated that they had diabetes on the qualifications page. This method would have resulted in a sample of 559 participants, or 13.42% (559/4165) of the original response pool. With a prevalence rate of 9.3% in the United States [38], 13.42% of the sample reporting diabetes is a larger percentage than one might expect in a national sample. However, this finding would not have been remarkable; it is consistent with the idea that people with diabetes might be drawn to a “Health Survey.”

#### The Remaining 252 Participants

Six percent of the initial pool of 4165 respondents made only a single attempt to take the survey and reported having diabetes (n=252). Demographic information for this subsample can be found in Table 2. Individuals ranged in age from 18-74 (mean=38.93, SD=13.6). With respect to diversity, the sample was predominantly non-Hispanic white, female, married, and had earned at least some college credit.

#### Disease Misrepresentation

As noted earlier, there were two initial qualifications pages on which participants indicated that they had diabetes and were 18 years of age or older. In addition, when participants provided informed consent, they clicked on a “submit” button to confirm that they were 18 years of age or older, with diabetes. Recall, however, that participants were also prompted about their disease status later in the actual survey with the question, “With which type of diabetes have you been diagnosed?” Of the 252 participants, 61 indicated they had type 1 diabetes (24.2%), 146 had type 2 diabetes (57.9%), 11 individuals did not know which type of diabetes they had (4.4%), and 3 individuals said they did not have diabetes (1.2%). Notably, 12.3% (31/252) of this subsample left this question blank. Therefore, it was necessary to exclude even more assuredly non-conscientious responses. Three people were excluded for indicating on this question that they did not have diabetes. One could also make an effective argument for excluding the 31 individuals (12.3%) who failed to answer this question.

#### Survey Completion Times

Pre-testing indicated that the survey would take approximately 25 min to complete. The average MTurk survey completion time was 12 min and 40 s (SD=19 min 3 s), ranging from completion in 8 s to 4 h and 45 min. Further analysis of the survey completion times indicated that 19.4% (49/252) completed the survey in less than 5 min. When our research team members were explicitly given instructions to barely skim the survey questions and answer randomly without thinking about their answers, the mean completion time was above 5 min. By all accounts, data from these 49 participants who completed the survey in less than 5 min (at the very minimum) should also be excluded from further analyses.

Fundamentally, in these instances we were examining the conscientious behavior of our participants. The Oxford English Dictionary defines conscientious as: “Wishing to do one's work or duty well and thoroughly.” Did the participants take the time to read the material carefully and thoroughly? Did they misrepresent their disease status? It is important to note that the small subsample of 252 individuals comprised participants we could not exclude from the sample for *not* being conscientious in their responses. Given that of these 252 participants, further examination indicated that at least 52 more responses should not be considered for evaluation, we could not, in good conscience, analyze any of the data for our original intent. Clearly, if one is not vigilant with survey design and Web-based parameter settings, the results can be disastrous. Therefore, our second survey was specifically designed to test a means by which to improve the likelihood of conscientious behavior, and to provide a way to detect cheating if it occurred.

### Study 2

#### Types of Trap Failures

Table 3 presents the descriptive data associated with the different types of trap failures. Trap failures were recorded for individuals who entered the incorrect date at the beginning of the survey, or who indicated that they, or a member of their family, had the fictitious disease of “hyperemblyopia.” Trap failures were also recorded for sex-specific trap questions (a male who indicated that he had been pregnant), as well as for individuals who alternately indicated that they had, and then did not have, diabetes.

**Table 2:** Demographic characteristics across each of the three samples.

Characteristics		Study 1, n (%); Original Sample	Study 2, n (%); MTurk	Study 2, n (%); Undergraduates
**Biological sex**					
	Male	95 (37.7)	138 (43.7)	67 (22.4)
	Female	153 (60.7)	176 (55.7)	232 (77.3)
	Did not specify	4 (1.6)	2 (0.6)	1 (0.3)
				
**Race**					
	Non-Hispanic white	172 (68.3)	244 (77.2)	222 (74.0)
	American Indian or Alaska Native	7 (2.8)	2 (0.6%)	3 (1.0)
	Asian American, Native Hawaiian, or other Pacific Islander	19 (7.5)	24 (7.6)	21 (7.0)
	Hispanic or Latino American	16 (6.3)	12 (3.8)	16 (5.3)
	Non-Hispanic black or African American	25 (9.9)	26 (8.2)	17 (5.7)
	Other	10 (4.0)	7 (2.2)	18 (6.0)
	Did not specify	3 (1.2)	0 (0.0)	3 (1.0)
				
**Marital status**					
	Single	62 (24.6)	150 (47.5)	291 (97.0)
	Married or partnered	119 (47.2)	115 (36.4)	7 (2.4)
	Divorced	38 (15.1)	42 (13.3)	1 (0.3)
	Separated	6 (2.4)	4 (1.2)	0 (0.0)
	Widowed	8 (3.2)	5 (1.6)	0 (0.0)
	Did not specify	19 (7.5)	0 (0.0)	1 (0.3)
				
**Educational level**					
	No High School Diploma	12 (4.8)	3 (0.9)	0 (0.0)
	High School Diploma, GED^a^ or Equivalent	30 (11.9)	36 (11.4)	72 (24.0)
	Some College Credit	85 (33.7)	97 (30.7)	208 (69.3)
	Associate’s Degree	21 (8.3)	39 (12.3)	11 (3.7)
	Bachelor’s Degree	62 (24.6)	114 (36.1)	7 (2.4)
	Master’s Degree	21 (8.3)	22 (7.0)	1 (0.3)
	Professional Degree	2 (0.8)	3 (1.0)	0 (0.0)
	Doctorate	2 (0.8)	2 (0.6)	0 (0.0)
	Omitted	17 (6.8)	0 (0.0)	1 (0.3)
**Total number of participants**		252	316	300

^a^GED: general education diploma.

**Table 3:** Different types of trap failures.

Trap category	Number of failures in MTurk sample	Number of failures in undergraduate sample	Total number of failures
Date (1 question)	19	10	29
Fictitious disorder 9 questions)	0	2	2
Sex-specific (13 questions)	7^a^	2	9
Diabetes (2 questions)	2	0	2
Survey completion time	13	5	18
Total	41	19	60

^a^This represents 3 participants with 1 failure and 2 participants with 2 failures.

**Table 4:** Percentage of trap failures.

Number of traps failed	MTurk, %	Undergraduate, %	Combined samples, %
0	88.0	95.0	91.4
1	11.1	4.0	7.6
2	0.9	0.7	0.8
3	0.0	0.3	0.2

#### Survey Completion Times

The time it took for participants to complete the survey was calculated for all items presented *after* the alert statement. The survey pretested at an average of just over 7 min (mean=7 min 14 s). Participant survey completion time ranged from 1 min and 24 s to 1 h 19 min (mean=5 min 39 s, SD=4 min 22 s). Univariate general linear modeling indicated that, on average, participants who received the alert took longer to complete the remainder of the survey (mean=5 min 47 s) than those who did not receive an alert (mean=5 min 32 s), but this difference failed to reach significance: *F*_1,610_=.40, *P*=.53, ƞ²=.001, power=.10. Notably, the effect size associated with the alert hovered near zero. On average, MTurk participants completed the survey 1 min and 37 s faster (mean=4 min 47 s) than did the undergraduate sample (mean=6 min 24 s): *F*_1,610_=16.97, *P*<.001 ƞ²=.027, power=.98.

For the purposes of trap failure, any person who exceeded three standard deviations above the mean time (18 min 45 s) received a trap failure notation. Given the large standard deviation associated with completion time (SD=4 min 22 s), using a similar three standard deviation rule below the mean was not sufficient and would have permitted the inclusion of a completion time of 0 s. Simply randomly completing the survey without reading the questions or the answers takes more than 2 min. Therefore, any person who took less than 2 min to complete the survey received a trap failure notation.

#### Trap Failure Rates

There were a total of 26 trap opportunities embedded within the survey (see Tables 3 and 4). Trap failure responses ranged from zero to three trap failures. The modal trap failure response was zero, while the mean was 0.092 (SD=0.32). There were only 60 trap failures in a context where the potential number of trap failures could have exceeded 16,000.

Univariate general linear modeling was then used to examine trap failure rates across the participant sample, biological sex, as well as within the alert statement manipulation. A significant difference emerged between the MTurk and undergraduate samples: *F*_1, 604_=4.33, *P*=.04, ƞ²=.007, power=.55. Although the overall magnitude of trap failures was actually quite low (mean=0.09), the MTurk sample had approximately twice as many trap failures (mean=0.12) than did the undergraduate sample (mean=0.06).

Moreover, although the presence of an alert resulted in fewer trap failures (mean=0.07) as compared with when no alert was present (mean=0.11), this difference failed to reach significance: *F*_1, 604_=2.5, *P*=.11, ƞ²=.004, power=.35. In addition, no significant differences emerged across sex, *F*_1, 604_=.62, *P*=.43, ƞ²=.001, power=.12.

### Sample Diversity

Chi-square analyses indicated that, when compared with the MTurk Master Worker sample (see Table 2), the undergraduate sample included more females (χ^2^_1_=31.9, *P*<.001), fewer individuals who were married (χ^2^_4_=188.4, *P*<.001), and, naturally, fewer individuals who had obtained a Bachelor’s degree or higher educational qualification (χ^2^_7_=189.5, *P*<.001). The samples did not differ with respect to race (χ^2^_6_=8.6, *P*=.20), but the MTurk sample (mean=37.67) was considerably older than the undergraduate sample (mean=19.37; *t*_598_=25.36, *P*<.001).

### Replicating Previous Data Trends

Several other studies have shown robust effects such that the perception that one could control disease onset would result in higher levels of perceived responsibility for disease onset, as well as higher ratings of anger and blame [37]. These findings were fully replicated within the present data. Multivariate general linear modeling revealed that participants who read scenarios in which the target acquired a disease through lifestyle choices rated the target higher in responsibility, anger, and blame (mean=3.15, 2.01, and 2.76, respectively) when compared with ratings for targets who were said to have acquired their disease through a genetic contribution (mean=1.44, 1.26, and 1.34, respectively): *F*_3, 596_=183.77, *P*<.001, ƞ²=.48, power=1.0. These ratings did not differ across sample, were not influenced by the presence of an alert, nor were they influenced by the type of disease presented in the scenario (diabetes or heart disease): *Fs*_3, 596_<1.78, *Ps*>.15, ƞ²<.009, power<.46.

## Discussion

### Study 1

We utilized MTurk to attract a very specific type of respondent; indeed, the data was gathered simply, effortlessly, and at an affordable total cost. However, with the initial discovery that nearly one-third of the responses represented duplicate IP addresses, the focus of the study was re-directed towards examining the quality of the Workers’ responses.

#### The Quality of Our MTurk Data

The survey had been launched without restricting it from being completed by two or more people at the same IP address. The justifications for this decision were as follows: (1) If more than one individual with diabetes resided at a particular household, we wanted the survey to be open to all members of the household, and (2) This was a 25-min survey paying only US $0.25.

With this in mind, lying about having diabetes for US $0.25 did not seem to be an advantageous decision. With respect to subversive activities, Berinsky et al use the same logic to suggest that “given the relatively low pay rate of our studies and the availability of other paid work, we do not believe our work is likely to encourage such behavior” [19]. Yet the sheer number of individuals who entered our study using duplicate IP addresses was unexpected, and, in retrospect, naïve. Because these IP addresses were presented within seconds of one another, the most plausible explanation is that these were Workers attempting to get past the qualifications page to receive compensation. It is essential that researchers properly utilize controls to protect against repeated access from a single IP address.

In addition, this particular study paid only US $0.25 for a lengthy survey. It is possible that this amount of money is not sufficient for participants to invest conscientiously in the work [39]. The survey description, however, included the appropriate time estimate for completion. Workers had the opportunity to simply avoid the survey given the explicit expectations, but some may have chosen, instead, to complete the work with low quality. High pay, however, may not mitigate these concerns because Chandler and Paolacci provide evidence that participants were even more likely to try to fraudulently enter a high-paying study (US $1.00) than a low-paying study (US $0.25) [40].

Another red flag was the extremely quick, virtually inhuman survey completion times. Maniaci and Rogge report that, in some types of studies, respondents demonstrating extraordinarily fast reaction times are simply and easily—and routinely—excluded from analyses [31]. In our case, that would be advisable because some of our responses were most likely responses from automated form-filling bots that have been programmed to complete Internet surveys. For example, in our study, 12 respondents logged response times of less than 60 s on a survey that had been pretested at 25 min. Moreover, our research team could not reproduce these speeds even when we tried, even by simply clicking each page without reading any portion of it. As a result of our experience, we believe it is essential for researchers to report survey completion time data as a perfunctory part of the publication process. In addition, adding a “CAPTCHA” would also be a recommended practice because it protects against bots by generating tests that humans, but not computer programs, can accomplish.

The subsample of 252 was comprised of participants we could not exclude from the sample for *not* being conscientious; however, it did not allow us to conclude that they *were* conscientious in their responses. Nor did it allow us to conclude that they were actually diagnosed with diabetes, despite their answers on the initial qualifications page. For example, some research suggests that the use of qualifications pages at the beginning of a survey is not optimal to study design. Chandler and Paolacci provide evidence that explicitly prescreening conditions can substantially increase fraudulent reports in an attempt to meet study qualifications [40]. The authors believe that the prescreen practice may lend researchers to be overly confident that the respondent is honestly reporting a particular characteristic or condition (eg, race or diabetes). These data are disturbing given that the basis for the interpretation of entire projects can rest on the supposition that a respondent possesses certain specific characteristics [25]. Chandler and Paolacci contend that these types of responses “create an obvious validity problem and may lead to erroneous conclusions about the population of interest” [40].

Instead, Chandler and Paolacci propose a pre-screen survey. Those individuals who acknowledge particular characteristics important to the study design in the initial pre-screen survey could then be invited back to the actual survey. Notably, Chandler and Paolacci believe that this could have the added benefit of enabling the recruitment of a more diverse pool of respondents [40].

Nonetheless, we must openly acknowledge that recommending that we must restrict duplicate IP addresses, that we cannot be confident that participants will be honest when answering qualification questions, that we should not use obvious screens, or that we must cross a particular threshold of monetary payments to get high-quality data affirms an underlying assumption that a substantial portion of MTurk Workers cannot be trusted to produce honest, high-quality data. Ipeirotis provides evidence that the majority of US MTurk Workers do not participate because the tasks are fun [41]; instead, 12% use it as a primary source of income, and money earned on MTurk is “...at least relevant to the vast majority of MTurk Workers” [17].

#### The Diversity of Our MTurk Data

Participant diversity was essential to the conduct of the present study. If we limit our attention to the 252 respondents that could not, a priori, be excluded from consideration, we find that the demographic composition is roughly similar to that reported in other MTurk samples. We report that 60.7% (153/252) were female, whereas Berinsky et al found that 60.1% were female [19]. Berinsky et al reported a mean age of 32.3 years, whereas our sample averaged nearly 39 years. A large portion of our sample reported being married (47.2%; 119/252). Berinsky et al reported that large percentages of MTurk Workers report never having been married and that they currently rent the home they are living in; however, we would also expect to see that trend in a college student sample.

With respect to the self-report of race or ethnicity, 83.5% of the Berinsky et al sample was white [19], whereas 68.3% (172/252) of our sample identified as Non-Hispanic white. The samples were comparable with respect to the proportion of Hispanic individuals, but our sample reported more than twice as many African Americans as Berinsky et al (9.9% vs 4.4%).

Ultimately, as we continue to applaud MTurk for its ability to secure a diverse sample, it is important to make a clear distinction between whether a sample is more diverse than some standard (eg, a college student population or adult convenience sample) and whether it meets the criteria for what would constitute a diverse sample in and of itself. For example, Behrend et al report a significant chi-square difference between their MTurk and undergraduate samples in terms of ethnicity [21]. Rather than being actually more diverse, one interpretation might be that the crowdsourced sample appears instead to be differently diverse, with more Hispanics but fewer African-Americans. The authors highlight that both samples are, nonetheless, predominantly Caucasian (82.20% and 79.78%, respectively). Likewise, in the current sample, we ultimately obtained data on only 77 individuals across several racial categories. We certainly did not meet any reasonable standard for what would constitute a truly diverse sample.

#### Summary

As problematic as these data were, they highlighted a very important question: Are there other researchers out there who have made similar mistakes? This could suggest two important potential outcomes: (1) Perhaps those Web-based responses made it through the publication process, or (2) Perhaps there are a lot of “file drawer” research studies out there that have quietly produced poor quality Web-based data, which is a methodological issue that needs to be openly discussed, debated, and formally addressed. This effort prompted the question: How can we *know* if our data is of sufficiently high quality unless we methodically test for it?

### Study 2

Clearly, as indicated in Study 1, if one is not vigilant with survey design and Web-based parameter settings, the results can be disastrous. Our second survey was specifically designed to test a means by which to increase the likelihood of conscientious behavior, to provide a way to detect cheating if it occurred, and ultimately, to encourage the purposeful reporting of such information by researchers during the journal review process. There are a number of techniques that help in keeping unconscientious responders out of surveys. For example, MTurk provides the opportunity to set high approval ratings and restrict samples to individuals who have completed a large number of successful HITs. Our first study was restricted to individuals who had a 90% approval rating or better, while our second study was restricted to elite Master Workers with an approval rating of at least 95%. Restricting to Master Workers, however, severely limits the pool numbers and, by its very nature, would likely result in a more homogenous group; this technique would further limit generalizability. Thus, some researchers simply use a 95% or greater approval rating, without the application of the Master Worker designation [40].

However, one must consider the accuracy and utility of approval ratings. Approval ratings must be used carefully. If you approve a Worker’s submission, the Worker gets paid. If you reject the Worker’s submission, payment is not made. Given Institutional Review Board protocols for conducting research with human subjects, it is likely that approval ratings are artificially inflated by some social science research projects. For example, it is quite common for research participants to be told that they may choose to skip questions they feel uncomfortable answering. At the same time, it would then be unethical for a researcher to reject a Worker’s submission for not being complete. While this may be irrelevant to individuals using MTurk for “work,” this is particularly tricky if you are conducting research on a sensitive health topic. The way around this, of course, is to present a consent form that fully and clearly articulates that (1) the Worker must answer every question in order to receive compensation and (2) a person will not receive compensation unless he or she answers each question adequately. It is likely that this practice could discourage respondents from participation. In support of the idea that approval ratings may be inflated, Kumar initially suggested using Workers with an approval rating greater than 95%, but less than one year later had increased his recommendation to greater than 98% [42].

Alternatively, one could improve data quality by improving conscientious responding *during* the administration. With this in mind, we empirically tested whether an alert would improve conscientious behavior. Not only did our alert have no impact on conscientious responding, the effect size was functionally zero. This outcome is potentially the byproduct of a ceiling effect of our highly conscientious sample; nonetheless, the present data suggest that an alert may not be the answer.

Finally, one can work to assure data quality *after the fact* by including traps to test for conscientious responding. We found that, although MTurk Master Workers were more likely to fail traps than the undergraduate sample, overall trap failure rates were remarkably low. We do not want to lose sight of what we believe to be exceptional performance on our survey by emphasizing that there were only 60 trap failures in a context where the potential number of trap failures could have exceeded 16,000. And, in fact, we were able to fully replicate an established finding in psychosocial health research.

One can also examine how long it took the respondents to complete the survey in comparison to a pre-testing standard [43]. One could exclude the responses from all participants who exceed three standard deviations from the mean completion time, as was done in the present study. However, speed is not a singularly effective determinant of unconscientious responders. Those who are unconscientious “speeders” could potentially manipulate their overall response time simply by spending some amount of time idling. Respondents could also be exposed to distractions such as phone calls or text messages that would affect response time. In addition, Clifford and Jerit provide evidence that respondent motives can significantly affect response time, which makes it difficult to determine the meaning that should be attached to response time [29]. Their data revealed a positive correlation between cheating and response time; students who were self-reported cheaters spent longer answering questions. Thus, unreasonably fast speeds could reflect a respondent not paying attention, while unreasonably slow speeds could be consistent with the idea that cheating was a function of searching outside sources for a “correct” answer to a knowledge question. Our study also showed that we can expect MTurk respondents to be significantly faster in completing a Web-based survey. This is likely due to their extensive experience with this medium. It certainly is a difficult balancing act when considering response time, given that faster speeds in an MTurk respondent result in financial gain.

Perhaps comprehensive examples for trap measures ought to be developed for researchers to implement in their Web-based surveys. For example, a trap question might ask the participant to select the word “cat” from a list of response options. In this case, it is not an attention check because the participant could continue to subsequent questions even if they answer incorrectly, but the researcher would know that the participant had not read the question carefully. However, even this recommendation that trap questions be routinely utilized within surveys may not necessarily assure conscientious responses. MTurk users are sophisticated and would quickly become aware of any specific trap questions that were recommended [25]. In fact, MTurk blogs exist where MTurk users routinely compare information about HITs; in this community, word can spread quickly. Most importantly, our embedded traps were specifically designed to camouflage within the cover story of our research. These questions were not salient, and this continues to be a very desirable design feature. Because this was a study, in part, about trap questions, a total of 26 potential traps were included. While we do not suggest that there is a need for that many trap questions in subsequent studies, we would suggest that at least some trap questions be included in every survey, and that they be fully and carefully camouflaged during the survey design phase.

When a survey response is flagged as being of poor quality, the data analysts must decide whether to keep or delete the data. Measuring response accuracy ultimately leads to a discussion on what to do with the data after poor-quality responses have been identified. Casler et al suggest that “...with thoughtful and creative manipulation checks in place, researchers usually can discard participants who have not taken the task seriously or who had insufficient skills to complete it correctly” [44]. This is not a universally accepted proposition as Chandler et al contend that researchers are overzealous when excluding participants [45]. Moreover, Oppenheimer et al, as well as Berinsky et al offer an interesting perspective by suggesting that removing inattentive performers skews the sample by removing a certain type of person [46,47]. For example, if people who are more educated pay more attention and pass more screens, then the resultant sample will be biased in favor of more educated respondents, potentially influencing validity. Thus, the paring down of a sample by discarding “certain” participants would seem to be a very slippery slope. Who, exactly, should be deleted, and how many participants can be deleted for viable use of a dataset? We do not pretend to offer a definitive solution, but we do believe that at least a reporting of this information should be a perfunctory part of the review process.

#### Limitations

##### Sample Diversity

We started this investigation with an attempt to achieve sample diversity. Compared to the MTurk sample, the undergraduate sample included more females, fewer individuals who were married, and, naturally, fewer individuals who had obtained a Bachelor’s degree or higher. The samples did not differ with respect to race. Overall, out of the 616 total participants recruited over a span of 15 weeks, only 121 self-reported a race other than Non-Hispanic white.

To mitigate health disparities, it is essential to study diverse samples. Some researchers suggest that one way to increase diversity representation in Web-based samples is to screen a large pool of MTurk Workers and then select a subset of participants who match desired sample characteristics [19]. Because of issues associated with access to computers and the Internet, however, this method will still ignore those of lower socioeconomic status that we often want as participants in health research. While MTurk samples may be ideal for work and specific types of research questions, these samples may not impact our ability to make a meaningful contribution to understanding how to reduce health disparities.

In essence, it does not appear, in any rendition, that our diversity goals were met. While published reports laud MTurk for its ability to foster a diverse sample, our studies highlight that while the sample could potentially be more diverse than a standard college student sample, our efforts did not lend themselves to meeting the criteria for what would constitute a diverse sample in and of itself. Our two studies provide support for continuing a meaningful mutualistic environment and presence in, for example, community centers and churches. Ultimately, improving open access to a wide range of study participants and funding cost-effective and cooperative efforts for a variety of health-relevant studies are ways to mitigate health disparities.

##### Recommendations

Above all, our recommendation is not to be complacent. With respect to diversity, it is imperative to find ways to expand minority recruitment efforts even in an Internet environment. With respect to data quality, we suggest that every posted survey include traps. Attention checks alone are not sufficient—they would serve as reminders during the survey administration, but would not assist in assessing the quality of the resultant responses. Notably, some researchers suggest that questions with factual answers should be avoided in the survey design phase, arguing that participants may be more likely to use the Internet to search for correct answers [32]. However, routinely avoiding factual answers would be a costly mistake because, by definition, we would be unable to *ever* assess the quality of the resultant data. Ultimately, in any self-report format, it is difficult to be certain that your participants have been conscientious in their responses. This is particularly the case with Web-based surveys, where a certain degree of trust is implicit in every administration. Of course, the same criticism would be made about any self-report measure, even if the participant is in the same room with the researcher.

### Conclusions

Published studies in the health domain are rapidly appearing that utilize MTurk participants. Some report the qualifications that were imposed on the participants during data collection or the quality control checks that were applied to the resultant data [48,49]. Others present insufficient detail or no information at all [50,51]. As a result, the reader does not know whether sufficient standards were applied and not reported, or not applied at all. Because some researchers have clearly experienced successful data collection on MTurk, while others report disastrous results, Kees et al recently identified that one essential area of research is the continued investigation of the types and magnitude of cheating behavior occurring on Web-based platforms [24]. These studies can contribute to this dialogue, and they alternately provide evidence of disaster and success. As a result of our experience, however, we would strongly suggest that standards be in place for publishing the results of Web-based surveys of health-relevant data, as an expanded version of the CHERRIES Checklist [52]. These standards should protect against publication of surveys that do not include suitable quality assurance tests built into the survey design, distribution, and analysis. We would recommend that specific information be reported, including the settings for the hosting platform, any filters that were applied, as well as the specific qualifications of the Worker. How much incentive was provided? And how did participants respond to embedded trap questions? It is essential that we create strict protocols for reporting quality checks of all data collected through Web-based research. Health-relevant research, in particular, cannot risk conclusions built on faulty data, and this should not be a file-drawer problem. We must scrutinize Web-based methodological techniques as we would any other paradigm.

## Abbreviations

AMT: Amazon Mechanical Turk
HIT: Human Intelligence Task
IP: Internet Protocol
MTurk: Amazon Mechanical Turk
NSSE: National Survey of Student Engagement
